# Biological significance of complex *N*-glycans in plants and their impact on plant physiology

**DOI:** 10.3389/fpls.2014.00363

**Published:** 2014-07-22

**Authors:** Richard Strasser

**Affiliations:** Department of Applied Genetics and Cell Biology, University of Natural Resources and Life SciencesVienna, Austria

**Keywords:** endoplasmic reticulum, Golgi apparatus, protein glycosylation, *N*-glycosylation, glycoprotein, *N*-acetylglucosaminyltransferase

## Abstract

Asparagine (*N*)-linked protein glycosylation is a ubiquitous co- and post-translational modification which can alter the biological function of proteins and consequently affects the development, growth, and physiology of organisms. Despite an increasing knowledge of *N*-glycan biosynthesis and processing, we still understand very little about the biological function of individual *N*-glycan structures in plants. In particular, the *N*-glycan-processing steps mediated by Golgi-resident enzymes create a structurally diverse set of protein-linked carbohydrate structures. Some of these complex *N*-glycan modifications like the presence of β1,2-xylose, core α1,3-fucose or the Lewis a-epitope are characteristic for plants and are evolutionary highly conserved. In mammals, complex *N*-glycans are involved in different cellular processes including molecular recognition and signaling events. In contrast, the complex *N*-glycan function is still largely unknown in plants. Here, in this short review, I focus on important recent developments and discuss their implications for future research in plant glycobiology and plant biotechnology.

## INTRODUCTION

**FIGURE 1:**
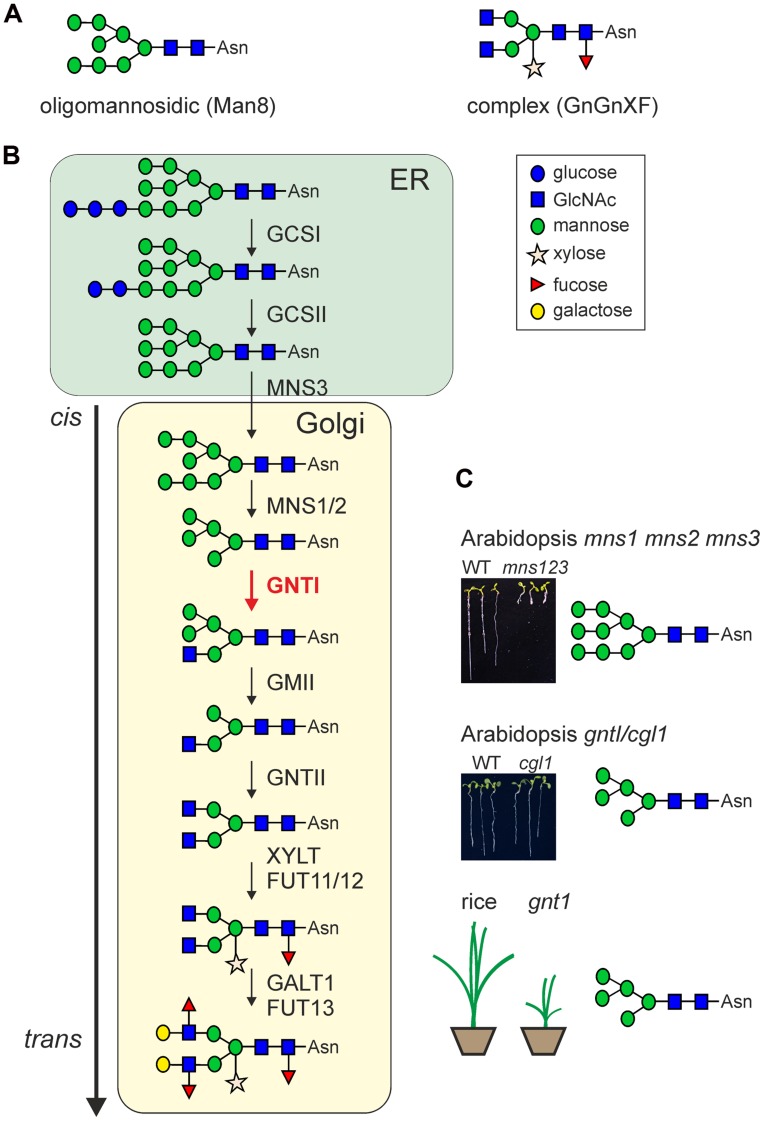
**(A)** Examples for two characteristic types of *N*-glycans linked to the Asn–X–Ser/Thr sequence of proteins: oligomannosidic (e.g., Man8) and complex-type (e.g., GnGnXF) *N*-glycans. **(B)** Possible route in the formation of complex *N*-glycans in plants. Upon transfer of the preassembled oligosaccharide, the first *N*-glycan trimming reactions are catalyzed by α-glucosidases I (GCSI)/ II (GCSII) and α-mannosidase 3 (MNS3). Complex *N*-glycan formation is initiated in the Golgi apparatus by β1,2-*N*-acetylglucosaminyltransferase I (GNTI, highlighted in red). MNS1/2, Golgi α-mannosidase I (two forms with largely redundant function are present in *A. thaliana*); GMII, Golgi α-mannosidase II; GNTII, β1,2-*N*-acetylglucosaminyltransferase II; XYLT, β1,2-xylosyltransferase; FUT11/12, core α1,3-fucosyltransferases (two forms with largely redundant function are present in *A. thaliana*); GALT1, Lewis-type β1,3-galactosyltransferase; FUT13, α1,4-fucosyltransferase. Structural analysis of *N*-glycans from different *A. thaliana* mutants and *in vitro* enzyme activity assays revealed that downstream of GNTI the substrate specificity of the processing enzymes is less stringent (Strasser et al., 2006, 2007b). Not shown: the possible removal of terminal GlcNAc residues by β-hexosaminidases (HEXO proteins) which generates paucimannosidic *N*-glycans in post-Golgi compartments or in the extracellular space (Liebminger et al., 2011). **(C)** The phenotypes of characteristic *N*-glycan-processing mutants are shown. While an *N*-glycan-processing defect *(mns1 mns2 mns3*) upstream of GNTI results in a severe root and shoot phenotype in *A. thaliana* (Liebminger et al., 2009), *cgl1* (or *gntI*, the allelic *A. thaliana* T-DNA knockout mutant) does not display any growth or developmental phenotype under normal growth conditions (von Schaewen et al., 1993; Kang et al., 2008). In contrast, rice *gnt1* displays a severe growth phenotype resulting in early lethality (Fanata et al., 2013). The major *N*-glycan structures of the mutants are indicated.

*N*-Glycosylation is a major co- and post-translational modification of proteins in all eukaryotes. It has been estimated that the majority of all secretory proteins are *N*-glycosylated (Apweiler et al., 1999). *N*-Glycosylation is initiated in the ER by transfer of a preassembled oligosaccharide (Glc_3_Man_9_GlcNAc_2_) precursor to asparagine residues within the sequence motif Asn–*X*–Ser/Thr (*X* represents any amino acid except proline) on nascent polypeptide chains. In addition, *N*-glycosylation at the unusual Asn–X–Cys site has also been described for some proteins (Matsui et al., 2011; Zielinska et al., 2012). Upon transfer of the oligosaccharide, the *N*-glycan is rapidly processed by highly specific α-glucosidases and α-mannosidases that remove terminal glucose and mannose residues, respectively. Incompletely trimmed *N*-glycans (Glc_0-3_Man_5-9_GlcNAc_2_) that contain different amounts of mannose residues (also called oligomannosidic *N*-glycans) are mainly found on ER-resident proteins (**Figure 1A**). The mannose trimming reactions are carried out by α-mannosidases (MNS1–MNS3) that act in the ER and Golgi (Liebminger et al., 2009). The Man_5_GlcNAc_2_ oligosaccharide, which is the final product of these early *N*-glycan-processing steps is used by GNTI as a acceptor substrate for the transfer of a single *N*-acetylglucosamine (GlcNAc) residue to the exposed α1,3-mannose of the *N*-glycan (Strasser et al., 1999). This enzymatic reaction is absolutely required for all further *N*-glycan modifications and results in the formation of complex *N*-glycans in the Golgi apparatus. In particular, GNTI generates the GlcNAc_1_Man_5_GlcNAc_2_ *N*-glycan that is further processed by Golgi-α-mannosidase II (GMII), GNTII, XYLT, and FUT11/12 (**Figure 1B**). All these enzymes are absolutely dependent on GNTI activity and reside in the *cis*/medial-Golgi apparatus of plants where they might form a multi-protein complex that could play a role for the organization of the glycosylation enzymes within the Golgi and subsequently also for the controlled processing of *N*-glycans (Schoberer and Strasser, 2011; Schoberer et al., 2013). GNTI is evolutionary highly conserved and present in land plants including mosses as well as in some microalgae (Strasser et al., 1999; Koprivova et al., 2003; Baïet et al., 2011). Due to its central function in initiation of complex *N*-glycan formation, GNTI controls the final *N*-glycosylation pattern on individual glycoproteins which can influence their biological function. XYLT and FUT11/12 attach β1,2-xylose and core α1,3-fucose residues, respectively, to different acceptor substrates and create common complex plant *N*-glycans like GlcNAc_2_XylFucMan_3_GlcNAc_2_ (GnGnXF, **Figure 1A**). Such complex *N*-glycans are not present in mammals and thus can elicit an unwanted anti-carbohydrate immune response when for example present on plant-produced recombinant glycoproteins (Bardor et al., 2003; Jin et al., 2008).

The final *N*-glycan modification steps take place in the *trans*-Golgi and are carried out by the Lewis-type β1,3-galactosyltransferase (GALT1) and the α1,4-fucosyltransferase (FUT13) which generate the Lewis a-trisaccharide [Fucα1-4(Galβ1-3)GlcNAc-R] on complex *N*-glycans (Strasser et al., 2007b). The Lewis a-type structures seem ubiquitous in the plant kingdom (Fitchette et al., 1999; Wilson et al., 2001), but they are only present on a small number of still widely unknown glycoproteins and the biological function of these large complex *N*-glycans remains to be shown.

Truncated *N*-glycans are generated by removal of terminal GlcNAc residues in post-Golgi compartments. These paucimannosidic *N*-glycans have been found on vacuolar and extracellular glycoproteins (Strasser et al., 2007a; Liebminger et al., 2011).

## THE FUNCTION OF OLIGOMANNOSIDIC *N*-GLYCANS

Early *N*-glycan-processing reactions mediated by α-glucosidase I and II are essential for *Arabidopsis* and presumably also for other plant species (Taylor et al., 2000; Boisson et al., 2001; Gillmor et al., 2002; Soussilane et al., 2009; Farid et al., 2011; Wang et al., 2014). The generated oligomannosidic *N*-glycans are implicated in folding of nascent polypeptides and play an important role during ER-quality control processes and ERAD of misfolded or incompletely assembled glycoproteins (Aebi, 2013). The overall principles of these processes are conserved in eukaryotes. Recent findings suggest that monoglucosylated *N*-glycans in the ER are important for association with the lectins calreticulin or calnexin also in plants. For example, the pattern recognition receptor EFR involved in innate immunity and a misfolded variant of the brassinosteroid receptor BRI1 displayed a selective interaction with the plant-specific calreticulin 3 (Jin et al., 2009; Li et al., 2009). Additional data suggest that *N*-glycans present on these heavily glycosylated leucine-rich repeat receptor kinases are subjected to re-glucosylation by the folding sensor UDP-glucose:glycoprotein glucosyltransferase (UGGT) and glucosidase-mediated de-glucosylation followed by a release from the calreticulin/calnexin quality control cycle (reviewed in Liu and Li, 2014; Tintor and Saijo, 2014). Moreover, specific mannose residues present on terminally misfolded glycoproteins play also a crucial role for the selective disposal via ERAD (Hong et al., 2009, 2012; Hüttner et al., 2012, 2014) and a complete block of mannose removal in the *Arabidopsis mns1 mns2 mns3* triple mutant causes also a severe root growth phenotype (**Figure 1C**; Liebminger et al., 2009).

## THE FUNCTION OF COMPLEX *N*-GLYCANS

In all higher eukaryotes, GNTI is the central enzyme that initiates complex *N*-glycan formation on secreted and membrane-bound proteins that are trafficking through the Golgi to their final destination. Early studies in mice revealed that GNTI is essential for the development of embryos (Ioffe and Stanley, 1994; Metzler et al., 1994), but cultured mammalian cells can survive in the absence of complex *N*-glycans (Stanley et al., 1975). More recent genetic approaches revealed that the structurally diverse complex *N*-glycans on mammalian proteins participate in many different biological processes and distinct alterations are often associated with diseases (Lowe and Marth, 2003; Dennis et al., 2009). *Drosophila melanogaster* deficient in GNTI activity are viable, but display distinct phenotypes like abnormal brain development and a reduced life span (Sarkar et al., 2006). *Caenorhabditis elegans* GNTI-null mutants develop normally but are more susceptible to bacterial pathogens (Schachter, 2010). Together these findings highlight the importance of complex *N*-glycan modifications in various organisms.

In spite of the fact that complex *N*-glycans are ubiquitously present in plants (Wilson et al., 2001), their biological function is virtually unknown. The first mutant lacking complex *N*-glycans was isolated more than two decades ago by EMS mutagenesis of *Arabidopsis* and subsequent screening for lines that lack β1,2-linked xylose and core α1,3-fucose residues (von Schaewen et al., 1993). The isolated *complex glycan 1* (*cgl1*) mutants displayed a defect in the formation of complex *N*-glycans due to a point mutation in the gene coding for GNTI (Strasser et al., 2005). Consequently, in *cgl1* all endogenous glycoproteins carry exclusively oligomannosidic *N*-glycans with Man_5_GlcNAc_2_ as predominant oligosaccharide. Remarkably, the *Arabidopsis cgl1* plants are viable, fertile and do not display any obvious phenotype under different growth conditions including heat (30°C) and cold (8°C) stress or increased light conditions (von Schaewen et al., 1993; **Figure 1C**). Related studies identified various other *Arabidopsis* mutants with distinct defects in *N*-glycan-processing steps downstream of GNTI. In line with data for *cgl1*, no clear growth or developmental phenotypes were observed for *Arabidopsis* mutants that produce hybrid structures (Strasser et al., 2006) or complex *N*-glycans devoid of β1,2-xylose and core α1,3-fucose residues (Strasser et al., 2004). In agreement with these findings, neither the complete elimination nor the overexpression of the Lewis a-type structures on complex *N*-glycans caused a substantial change in *Arabidopsis* growth or development when grown under long day conditions (16 h-light/8 h-dark) at 22°C (Strasser et al., 2007b). Up to now, the only evidence for a biological function of complex *N*-glycans in *Arabidopsis* was found when *cgl1* and other mutants were subjected to osmotic and salt stress (Kang et al., 2008). Reduced root growth on media containing high NaCl concentrations indicated that complex *N*-glycans are implicated in tolerance to salt stress. However, a deeper understanding of complex *N*-glycan function in *Arabidopsis* and studies that associate distinct complex *N*-glycan structures on individual glycoproteins with the enhanced salt sensitivity are completely missing.

Based on the aforementioned studies, it has been suggested that *N*-glycan processing in the Golgi is dispensable for the normal development of plants and plays only a role under certain stress conditions. A recent study by Fanata et al. (2013) challenges our current view and provides strong evidence that complex *N*-glycans play indeed an essential role in some plant species. A homozygous *Oryza sativa* line (*gnt1*) with a T-DNA insertion in the single rice *GNTI* gene was identified that completely abolished *GNTI* mRNA expression. As a consequence of missing *GNTI* transcripts and in accordance with the central function of GNTI in the formation of complex *N*-glycans, the rice *gnt1* mutant displayed only oligomannosidic *N*-glycans with approximately 75% Man_5_GlcNAc_2_ structures. Almost the same amounts of Man_5_GlcNAc_2_ *N*-glycans were found for *Arabidopsis cgl1* (von Schaewen et al., 1993; Strasser et al., 2005). However, in marked contrast to *Arabidopsis cgl1*, a severe phenotype with arrested seedling development and lethality before reaching the reproductive stage was reported for rice *gnt1* (Fanata et al., 2013; **Figure 1C**). In addition, rice *gnt1* plants displayed defects in cell wall composition and cytokinin insensitivity. Although the final confirmation that the observed severe phenotypes are indeed linked to defects in *gnt1* is missing as the cytokinin defect caused problems with complementation of the *gnt1* plants, all other data are convincing and indicate that complex *N*-glycans are essential in some plant species. How can we explain this discrepancy between *Arabidopsis* and rice? Based on data from total *N*-glycan analysis and annotation of the rice genome, it is quite likely that the *N*-glycan-processing steps in the Golgi are very similar between the two species (**Figure 1B**). However, there might be subtle differences in cell-/tissue-specific expression of certain *N*-glycan-processing enzymes that might have been missed by total *N*-glycan analysis from whole plant organs. Interestingly, the rice genome contains more than one glycosyltransferase with homology to *Arabidopsis* GALT1 (Strasser et al., 2007b) and it seems that the formation of Lewis a-type structures occurs more frequently in rice than in *Arabidopsis* (Léonard et al., 2004; Strasser et al., 2004, 2007b). The rice GALT1 homologs belong to Carbohydrate-Active enzyme glycosyltransferase-family 31, which contains a large number of enzymes with quite diverse functions (Strasser et al., 2007b; Basu et al., 2013). These GALT1 candidates have not been characterized and in the absence of data from plants devoid of Lewis a-type structures, their contribution to the development of rice remains an open question. Moreover, *N*-glycosylation defects are generally pleiotropic and affect numerous secretory as well as membrane-anchored proteins. Consequently, the observed phenotype in rice *gnt1* could arise from several different glycoproteins that are dysfunctional in the absence of Golgi-mediated *N*-glycan processing. As rice *gnt1* displays reduced cellulose contents, glycoproteins involved in cellulose biosynthesis could be affected (Fanata et al., 2013). While impaired *N*-glycosylation or *N*-glycan processing has also been linked to changes in cellulose contents in *Arabidopsis* (Burn et al., 2002; Gillmor et al., 2002; Zhang et al., 2009) *gntI/cgl1* does not contain significantly altered cellulose contents compared to wild-type *Arabidopsis* (Kang et al., 2008). Recently, it was also shown that the heavily glycosylated endoglucanase KORRIGAN1, whose enzymatic activity is important for efficient cellulose formation, does not need complex *N*-glycans for its function (Liebminger et al., 2013).

Based on the detected cytokinin insensitivity it was speculated that members of the cytokinin-receptor family are *N*-glycosylated and their function might be impaired in the rice *gnt1* line (Fanata et al., 2013). These histidine sensor kinases contain an extracellular domain of approximately 280 amino acids with putative *N*-glycosylation sites (Caesar et al., 2011; Steklov et al., 2013). The degree of *N*-glycosylation and the *N*-glycan structures of cytokinin receptors are not very well known, but for *Arabidopsis* AHK3 *N*-glycosylation could be shown by transient expression in tobacco (Caesar et al., 2011). Notably, in *Arabidopsis* as well as in maize these receptors were primarily found in the ER implying that cytokinin binding takes place in this compartment (Caesar et al., 2011; Lomin et al., 2011; Wulfetange et al., 2011). If so, then Golgi-processed complex *N*-glycans are very likely not present on cytokinin receptors and consequently these receptors are not directly affected in GNTI-deficient rice.

## IMPLICATIONS FOR PLANT GLYCOBIOLOGY

To understand the mechanisms underlying the observed defects in rice *gnt1* and compare them with data from other plants species a number of key experimental approaches have to be explored: (i) It is very important to isolate other rice *N*-glycan-processing mutants to pin down the complex *N*-glycan structure or individual sugar residue that is crucial for the growth and development of rice. (ii) There is an urgent need for high-throughput glycoproteome approaches that enable the isolation of a large number of glycoproteins and mapping of the corresponding *N*-glycan structures from different plant species. Advances in this field will be crucial for structure–function analysis and identification of target glycoproteins. Plant *N*-glycoproteome studies have been reported recently (Zhang et al., 2011; Zielinska et al., 2012; Song et al., 2013), but compared to other posttranslational modifications these approaches are still too limited (Albenne et al., 2013). (iii) Information on regulation of glycosylation enzymes as well as information on cell-type or organ-specific occurrence of certain glycan structures is almost completely missing. Tools that have been used for the cell-type-specific analysis of protein expression (Petricka et al., 2012) should also be applied to unravel the *N*-glycoproteome in different plant species. (iv) Up to now, null mutants devoid of *N*-glycan processing have been characterized from *Arabidopsis* and rice, but information on the significance of *N*-glycosylation and complex *N*-glycan formation in other vascular plants is missing. Together, the highlighted experimental approaches will enable us to decode the biological function of the so far largely unknown complex *N*-glycan modifications like the attachment of β1,2-xylose, core α1,3-fucose, and the formation of the Lewis a-type structures.

## IMPLICATIONS FOR PLANT BIOTECHNOLOGY

Plants are emerging hosts for the manufacturing of valuable recombinant proteins. Recently, the first plant-produced recombinant biopharmaceutical, a recombinant human glucocerebrosidase, has been approved for enzyme replacement therapy in humans and is commercially available in the United States (Grabowski et al., 2014). Many biopharmaceutical proteins like human immunoglobulins or hormones are glycosylated and the composition of the glycans very often affect protein–protein interactions leading to altered efficacies of the recombinant drugs or unwanted side-effects like fast clearance from the blood or increased immunogenicity. Consequently, for the pharmaceutical industry as well as for structure–function studies, there is a growing demand to modify and control protein glycosylation of expression hosts. The ultimate aim of these approaches is the production of recombinant glycoproteins with defined and homogenous glycan structures (Rich and Withers, 2009; Dalziel et al., 2014). Developments during the last 10 years have shown that plants are amenable to glyco-engineering and capable of producing valuable recombinant glycoproteins with defined human-like structures (Castilho and Steinkellner, 2012; Nagels et al., 2012; Bosch et al., 2013). The absence of any growth phenotype in *Arabidopsis cgl1* laid the foundation for *N*-glycan engineering of other species like *Nicotiana benthamiana* and *Lemna minor* as well as of rice suspension cells (Cox et al., 2006; Strasser et al., 2008; Shin et al., 2011). In these studies, gene silencing of XYLT and FUT11/12 was used to eliminate the non-human and potentially immunogenic β1,2-xylose and core α1,3-fucose residues from complex *N*-glycans of recombinant proteins. Overall, these glyco-engineering efforts were quite successful, but the plants still produced low amounts of complex *N*-glycans like GnGnXF. A detailed characterization of null mutants for XYLT and FUT11/12 will reveal whether these and other plant species tolerate the absence of β1,2-xylose and core α1,3-fucose residues on endogenous glycoproteins during their whole life cycle. In addition, further studies are necessary to investigate in detail the consequences on growth, development, reproduction and stress response of stable engineered plants that carry human-type complex *N*-glycan modifications. So far, these knock-in approaches were limited to a small number of plant species and mainly to stable expression of single mammalian glycosyltransferases (Bakker et al., 2001; Rouwendal et al., 2007; Castilho et al., 2008; Sourrouille et al., 2008; Frey et al., 2009; Nagels et al., 2011). In contrast, most of the more advanced glyco-engineering approaches that require the concerted action of several mammalian enzymes were done by simultaneous transient expression of whole glycosylation pathways (Castilho et al., 2010, 2012, 2013). The stable expression of the proteins and enzymes involved in multi-step *N*-glycan processing like the formation of highly sialylated complex *N*-glycans without any negative effects on plant growth and development remains to be shown.

In the light of the recent findings from rice, glyco-engineering in some plant species might require new strategies and implementation of more elaborate tools to overcome adverse phenotypes linked with extensive *N*-glycan remodeling. In terms of plant glycobiology, the new findings from rice open the door for an exciting new era.

## Conflict of Interest Statement

The author declares that the research was conducted in the absence of any commercial or financial relationships that could be construed as a potential conflict of interest.

## ABBREVIATIONS

EMS: ethyl methanesulfonate
ERAD: ER-associated degradation
GNTI: β1,2-*N*-acetylglucosaminyltransferase I
FUT11/12: core α1,3-fucosyltransferase
XYLT: β1,2-xylosyltransferase
